# The Human Teeth

**Published:** 1866-11

**Authors:** John Allen


### THE HUMAN TEETH.

BY PROFESSOR JOHN ALLEN

MANY suppose that the human teeth did not formerly decay as in the present age, and that the dental art is of recent origin; but, in looking over the history of dental surgery, we trace it back more than four hundred years before Christ.

Hippocrates and Herodotus were among the Greek writers upon this subject. And from among the Romans, we have also the writings of Pliny, Martial, Horace, Celsus, and others, which reflect much light upon this branch of surgery; and although as a science it was then in a rude state, yet there were those who devoted their exclusive attention to the teeth at that time. Good gold fillings have recently been found in the teeth of mummies, which must have been inserted more than two thousand years ago.

Hippocrates informs us, that the loss of the natural teeth was supplied with that of artificial, made of bone or ivory, secured in the mouth by means of ligatures (made of flax, silk, gold or silver wire), tied to other remaining teeth in the mouth. Human teeth were also used and fastened in a similar manner. About one hundred and fifty years after Christ, Galen wrote a much better work upon this subject than any of his predecessors, yet very little advancement had been made during the previous five hundred years.

During the next fourteen hundred years, various authors wrote upon this subject, among whom were Ætius, Phazes, Albucasis, Vesalius, Eustachius, and others, whose writings set forth nearly the same modes of practice, as those adopted by the Greeks and Romans.

In fifteen hundred and seventy-nine, Pakie, a celebrated French surgeon, wrote a very correct essay upon the teeth. He enjoyed a great medical reputation, and was appointed surgeon in ordinary to Henry II., which office he held under three succeeding kings.

His cure for the toothache was to thrust a hot wire into the roots, or make an application of the oil of vitriol. He also taught the doctrine of transplanting teeth, which was done by extracting sound teeth from the mouth of one person, and inserting them in that of another of higher rank, whose teeth were decayed or uncomely. In this operation, the decayed teeth are first extracted; immediately after which the sound teeth are removed (generally from the mouth of a servant), and inserted while warm and fresh into the sockets just vacated for their reception. If this operation is well performed, under favorable circumstances the teeth and sockets in a few weeks become united and remain firm for many years. But this method has now become obsolete, and also that of tying artificial teeth with strings or wire.

Artificial teeth made of bone or ivory, were objectionable on account of their unnatural appearance, their liability to rapid decay in the mouth, and consequent tendency to become offensive. These objections led to the introduction of porcelain or incorruptible teeth, which were first conceived by Duchateau in seventeen hundred and seventy four, but not being a dentist, he was unable to carry out his theory practically, although he made some specimens that were capable of being worn, for which the Academy of Medicine of France granted him the honor of a seat. M. de Chemant, a practical dentist then in Paris, took up the idea where Duchateau had left it, and finally succeeded so well that he obtained a patent some twelve years after from Louis Sixteenth.

Other French dentists have also contributed much to the development of this important feature in dental practice. But to the Americans, is accorded the honor of having attained the highest degree of perfection in this branch of dentistry.

The usual mode by which porcelain teeth are now manufactured consists in combining certain mineral substances, such as quartz, feldspar, Kaolin clay, &c., in due proportions, which are ground, mixed, and rendered plastic by the addition of water, then moulded or carved to represent the teeth, and solidified in a furnace with intense heat.

The different tints and colors are produced by the use of metallic oxides; teeth are made without representing the gums and many are tipped with gum color upon each tooth. Two or more teeth are sometimes connected; these are called block teeth; metallic plates are usually employed as a base upon which the teeth are set. They are formed of gold, platinum, paladium, silver and (recently) aluminum, and sometimes a compound of metals; vulcanized rubber and gutta percha have also been used of late, for temporary work, the practical result of which remains to be seen.

Of all the various methods which have been employed for the construction of artificial dentures, that which combines the greatest advantages, and approximates the nearest to Nature, will command the largest share of public favor; it should display teeth of a perfectly natural form, tone, and truthful expression, embellished with an artificial gum, without seam or crevice, securing perfect cleanliness, exhibiting the roof and ruga of the mouth with all the delicate shades and tints peculiar to those of Nature, every tooth seeming to have grown out of the natural gum. By this method, the natural form and expression of the mouth and face can be restored, by raising the sunken muscles and sustaining them in their natural position, thus producing symmetry of form in the waning cheek and youthfulness of look.

---

The attention of the reader is invited to the above article on the teeth by a gentleman who is an honor to the Dental Profession. Those who are advanced in life and whose teeth are defective, may feel assured that artificial teeth can be so adjusted for practical purposes as to perform well the offices of the natural tooth in mastication, and this is specially important as a means of preparing the food for the more easy operation of the fluids which are provided for converting the food into ailment for the body; for the want of a proper and sufficient mastication has laid the foundation to multitudes of a life of suffering and invalidism.

